# Recent Developments in the Design and Fabrication of Electrochemical Biosensors Using Functional Materials and Molecules

**DOI:** 10.3390/bios13040424

**Published:** 2023-03-27

**Authors:** K. Theyagarajan, Young-Joon Kim

**Affiliations:** Department of Electronic Engineering, Gachon University, Seongnam 13120, Republic of Korea; thektr@gmail.com

**Keywords:** modified electrode, fabrication strategy, electrochemical immunosensor, aptasensor, disposable sensor

## Abstract

Electrochemical biosensors are superior technologies that are used to detect or sense biologically and environmentally significant analytes in a laboratory environment, or even in the form of portable handheld or wearable electronics. Recently, imprinted and implantable biosensors are emerging as point-of-care devices, which monitor the target analytes in a continuous environment and alert the intended users to anomalies. The stability and performance of the developed biosensor depend on the nature and properties of the electrode material or the platform on which the biosensor is constructed. Therefore, the biosensor platform plays an integral role in the effectiveness of the developed biosensor. Enormous effort has been dedicated to the rational design of the electrode material and to fabrication strategies for improving the performance of developed biosensors. Every year, in the search for multifarious electrode materials, thousands of new biosensor platforms are reported. Moreover, in order to construct an effectual biosensor, the researcher should familiarize themself with the sensible strategies behind electrode fabrication. Thus, we intend to shed light on various strategies and methodologies utilized in the design and fabrication of electrochemical biosensors that facilitate sensitive and selective detection of significant analytes. Furthermore, this review highlights the advantages of various electrode materials and the correlation between immobilized biomolecules and modified surfaces.

## 1. Introduction

Early diagnosis of diseases and disorders plays a vital role in safeguarding mankind against diseases, from common disorders to life-threatening situations, by detecting them at the initial stages. In addition, the timely detection of these diseases simplifies the medical treatment process, reducing the overall cost and treatment time [1,2,3,4]. Moreover, early treatment might decrease or control the disease growth rate and reduce further spreading to other parts of the body or other human beings [5,6,7]. Additionally, for some disorders, merely adopting a change in lifestyle helps the patients to overcome them over a period. Even though new medicines and treatment processes are being discovered every other day, many diseases still pose serious threats to all living beings. On the other hand, industrialization and population growth necessitate the production of various products in large quantities, from toilet paper to textiles, agricultural commodities to aerospace products, and potable water to rocket fuel, which involves numerous chemicals and liberates various waste products into the environment and water bodies. These waste products not only affect the environment but also causes various health hazards to all living beings [8,9,10,11]. Hence, these chemicals and other waste products need to be treated and eliminated or converted to other non-hazardous products; in order to achieve this, they need to be identified and quantified [12,13,14]. Therefore, it is indispensable to detect and quantify various significant analytes, from biofluids to environmental samples, in order to prevent or eliminate various diseases and disorders [15,16].

There are various sophisticated technologies and methodologies, such as high-pressure liquid chromatography, mass spectrometry, chemiluminescence, capillary zone electrophoresis, fluorimetry, calorimetry, electrochemical sensors, biosensors, DNA microarrays, PCR, ELISA, and other immunosorbent assays, being used for the detection and quantification of target analytes. However, most of these techniques are expensive and time-consuming, and require skilled manpower, tedious sample pretreatment, and huge instrumentation setup [17,18,19,20,21,22,23]. Among them, the electrochemical biosensor is the most preferred technology due to its robustness, simplicity, rapidity, portability, cost-effectiveness, ease of handling, high sensitivity, and selectivity toward target analytes, in addition to reliable and reproducible responses [24,25,26,27,28]. In general, electrochemical sensing was performed in a three-electrode cell system composed of a modified working electrode (WE), reference electrode (RE), and counter electrode (CE). Some of the commonly used WEs are glassy carbon electrodes (GCEs), gold electrodes (AuEs), graphite electrodes (GREs), screen-printed carbon (SPCEs) or gold electrodes (SPGEs), carbon paste electrodes (CPEs), and indium–tin oxide electrodes (ITOs). The commonly used REs are aqueous silver/silver chloride (Ag/AgCl filled with 1 M or 3 M KCl), printed Ag/AgCl, and saturated calomel electrodes (SCE). Common CEs are Pt wire, Pt spiral/coil, Pt mesh, and printed Pt electrodes. The WEs can be modified by various methods, such as simple drop casting, self-assembling, dip coating, electrodeposition, composite formation, spray coating, or even printing [29,30,31,32]. Depending upon the nature of the modifying material and its intended application, a suitable fabrication methodology can be chosen for the modification. The most crucial part of the fabrication of an electrochemical biosensor is the stable and effective immobilization of the desired biomolecules on the modified surface, because the biomolecules are highly sensitive, delicate to handle, and get denatured easily [33,34]. Furthermore, the active centers are deeply buried inside and are surrounded by a thick non-conducting peptide layer [35,36]. Therefore, only a suitable choice of material could stably immobilize these biomolecules and access their active centers without damaging their secondary structures. To address this, researchers have developed various biocompatible and conductive materials in order to improve and enhance the electrocatalytic performance of these immobilized biomolecules, which include metal nanoparticles, carbon nanomaterials, organic frameworks, ionic liquids, conducting polymers, quantum dots, nanodots, etc., and still the search continues [37,38,39,40]. As a result, thousands of biosensor platforms are being published every year. Therefore, we sought to write a collective report on recently developed biosensor platforms and detail their fabrication strategies. Furthermore, we have elaborated on the advantages of various materials and their sensible modifications towards the construction of efficient biosensors. In addition, this review stipulates the indispensable attributes of perennial biosensor platforms. We believe that the information furnished in this review would benefit the researchers working in this area, and serves as a road map for the design and fabrication of impeccable biosensors.

## 2. Various Nanomaterial-Modified Electrodes

**Figure 1:**
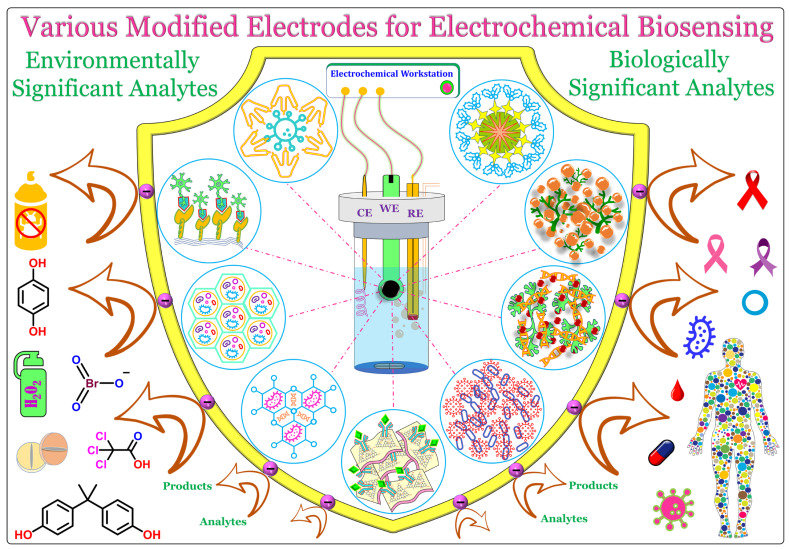
A conventional three-electrode setup (CE—counter electrode; WE—working electrode; RE—reference electrode) and biosensors fabricated using various functional materials and molecules (functionalized carbons and dendrimers, quantum dots and metal nanoparticles-decorated 2D materials, metal and covalent organic frameworks, porous materials and functionalized ionic liquids) for the electrochemical biosensing of biologically and environmentally significant analytes.

Nanomaterials (NMs) are materials ranging in size from 1 to100 nm. NMs have superior optical, physical, chemical, and electrochemical properties compared to their bulk. These properties can be tuned or tailored by altering the shape, size, and constituents of NMs. The following are some recently employed NMs and their hybrids in the design and fabrication of electrochemical biosensors. Figure 1 shows the general representation of a common three-electrode system, comprising a working electrode (WE), reference electrode (RE), and counter electrode (CE), and a graphical illustration of various biosensors fabricated using different electrode materials (functionalized carbons and dendrimers, quantum dots and metal nanoparticles-decorated 2D materials, metal and covalent organic frameworks, porous materials, and functionalized ionic liquids) for the biosensing of significant analytes.

### 2.1. Carbon-Based Nanomaterials

#### 2.1.1. Carbon Black

Carbon black (CB) is the thermal decomposition product obtained as a result of the incomplete combustion of various oil products. It is mainly composed of carbon and hydrogen, and some other elements used are nitrogen, sulfur, and oxygen [41]. It has various advantages, such as low cost, large specific surface area, good electrochemical properties, ease of functionalization, as well as excellent thermal and electrochemical stability. Therefore, it is one of the most commonly used electrode modifiers in the design and development of electrochemical sensors and biosensors. A catechol biosensor was fabricated using functionalized CB (fCB) and a dihexadecylphosphate (DHDP) modified electrode immobilized with tyrosinase (TYR) [42]. The functionalization of CB increased the oxygen-containing functional groups on the surface, which improved its wettability and electron transferability 100-fold compared to unfunctionalized CB. The GCE was initially modified with a mixture of fCB and DHDP, then TYR was drop-casted along with bovine serum albumin (BSA) and glutaraldehyde (Gld). The developed biosensor showed an excellent electrochemical detection of catechol in phosphate buffer (pH 7.5) with a lowest limit of detection (LOD) of 87 nM. Recently, a label-free *Escherichia coli* (*E. coli*) O157:H7 biosensor was developed using CB and carboxyl-functionalized graphene oxide (CGO) modified with phage EP01 [43]. In this case, the EP01 was covalently immobilized over the CGO/CB via 1-ethyl-3-(3-dimethylaminopropyl)carbodiimide/N-hydroxysuccinimide (EDC/NHS) coupling and the obtained biocomposite was immobilized over a clean GCE to obtain the desired biosensor. Finally, the fabricated biosensor was incubated with different concentrations of GXEC-N07 in order to bind it with EP01. The proposed biosensor exhibited a linear concentration range from 10^2^ to 10^7^ CFU mL^−1^ in 0.1 M KCl containing 5 mM of potassium ferricyanide. Subsequently, it was employed for the detection of GXEC-N07 in raw pork and fresh milk samples. Melios and colleagues developed an all-carbon-based flexible, non-invasive biosensor for glucose detection using glucose oxidase (GOD) immobilized over CB-decorated conductive carbon ink-modified polyimide substrate [44]. GOD was drop coated over the modified surface and covered with a layer of Nafion (Nf) solution. This developed sensor selectively sensed glucose in phosphate buffer (pH 7.4). However, the developed biosensor could not reach a sufficiently low LOD value in comparison with other reported biosensors. In another attempt, a magneto-immunosensor was developed for the detection of aflatoxin B1 using a horseradish peroxide (HRP) immobilized CB-modified screen-printed carbon electrode (SPCE) [45]. Based on this, a smartphone-based point-of-detection biosensor was fabricated and the sensing was carried out using a homemade open-source android application called AflaESens. Subsequently, the AlfaESens sensor was utilized for the analysis of aflatoxin B1 spiked in various corn samples.

#### 2.1.2. Graphene and Its Derivatives

Graphene (GR), the thinnest material utilized so far, is made of a single layer of carbon atoms arranged in a 2D honeycomb lattice structure with sp^2^ hybridization. GR is an exfoliated monolayer of graphite and has only a 1-atom thickness [46]. GR is an electrocatalytically active and transparent material with various advantages, such as high mechanical strength, excellent conductivity due to extended π conjugations, high thermal and chemical stability, large surface area, exceptional biocompatibility, and so forth. However, isolating a single layer of GR is highly complicated, as the layers are often agglomerate with one another. Due to its significant advantages in various fields, researchers have developed many derivatives of GR; some of them are few-layered GR (FLGR); GR nanosheets (GRNS); GR nanoribbons (GRNR); graphene oxide (GO); and thermally, chemically, and electrochemically reduced GO derivatives (TRGO/CRGO/ERGO) [47,48]. Among them, GO and RGO are mainly studied due to their unique physical and chemical properties, stability, and ease of synthesis. In electrochemical sensors, GO and RGO are the most preferred electrode materials and modifiers due to their excellent conductivity, electrochemical stability, exceptional support behavior, hydrophilicity, biocompatibility, rigidity, etc. Furthermore, the presence of oxygen-containing functional groups in GO and RGO allows them to chemically couple with various redox moieties and biomolecules, which enhances the stability and electrochemical activity of the developed system. Furthermore, the properties of GR and its derivatives can be improved or customized, depending upon the need, by doping them with various hetero atoms or metal atoms, depositing nanoparticles, imparting functional groups, or creating defects via etching [49,50].

Parlak and colleagues developed an anionic surfactant-functionalized GR for the immobilization of the enzymes cholesterol oxidase (ChOx) and cholesterol esterase (ChEs), as well as gold nanoparticles (GNPs), and fabricated biosensors for cholesterol and hydrogen peroxide (H_2_O_2_) [51]. The surfactants altered the electrostatic charges of GR, which facilitated the immobilization of biomolecules and GNPs by self-assembly on the GR surface. The developed biosensor displayed low detection limits of 25 nM and 50 nM for H_2_O_2_ and cholesterol, respectively, in 0.1 M phosphate buffer (pH 7.4). The proposed sensor has various advantages, such as a large surface area, excellent electron transferability, and good biocompatibility due to the presence of GR and GNPs in the biosensor platform. In another study, a GR-GNPs nanocomposite was designed and developed for the immobilization of the TYR enzyme and a bisphenol A (BPA) biosensor was fabricated [52]. Here, the agglomeration of GR sheets was prevented by incorporating GNPs in between the layers of GR, while GNPs were stabilized by GR sheets. The biosensor was fabricated by drop casting the nanocomposite containing optimized quantities of GR, GNPs, TYR, and chitosan (CS). The developed sensor exhibited a linear detection range of 2.5 nM to 3 µM in 0.1 M phosphate buffer (pH 7) and was also employed for the detection of BPA in real samples, such as milk cartons and disposable plastic cup samples. Kong and colleagues developed a hybrid platform using in situ direct electrochemical exfoliation of graphite to GR in the presence of hemin and SWCNT for the electrochemical sensing of H_2_O_2_ [53]. This developed hybrid system electrochemically reduced H_2_O_2_ at a very low potential of −0.2 V and exhibited a linear concentration range from 0.2 µM to 0.4 mM with a LOD of 50 nM in 0.1 M phosphate buffer (pH 7.4). Subsequently, it was employed for the detection of H_2_O_2_ spiked in beverages, such as jasmine, ice red and green teas, as well as orange and grape juice samples. In another attempt, tin oxide quantum-dots-functionalized 3D GR nanocomposite (QDGR) was developed for the immobilization of the myoglobin (Mb) enzyme and fabricated an electrochemical biosensor for sensing trichloroacetic acid (TCA) [54]. The biosensor was fabricated by drop casting QDGR over a carbon ionic liquid electrode (CILE), then Mb was coated and finally covered with a layer of CS to obtain Mb/QDGR/CILE. The direct electrochemistry of Mb was successfully achieved on the newly fabricated sensor due to the improved conductivity and biocompatibility offered by QD, GR, and CILE. Furthermore, the sensor displayed an excellent electrocatalytic reduction of TCA with a LOD of 0.35 mM in nitrogen-saturated phosphate buffer (pH 7.0). However, the LOD of the developed sensor was higher than other reported biosensors. Furthermore, it was employed for the detection of TCA spiked in medical facial peel solutions, which showed acceptable recoveries.

Poletti and colleagues developed a novel platform for the sensing of glucose and lactate in sweat using a PB- and CS-functionalized GO-based sensor [55]. GOD and lactate oxidase (LOx) were immobilized over the developed PB/CS-GO platform. This developed sensor showed excellent electrocatalytic activity towards glucose and lactate over an upper limit linearity of 3.8 mM and 50 mM, respectively, with a limit of quantifications (LOQ) of 32 nM and 68 nM, respectively. Subsequently, an electrochemical device was constructed for the simultaneous determination of glucose and lactate in body sweat. In another study, a nanocomposite made of GO-covered GRQDs was developed for the immobilization of dsDNA and employed for the sensing of dimethoate (DMT) [56]. Initially, GRQDs were electrodeposited over GCE, and then GO was drop cased to obtain the GO@GRQDs nanocomposite. Later, dsDNA was coated over the modified electrode by applying a constant potential to form the desired biosensor. These fabricated biosensors showed excellent electrocatalytic activity towards DMT over the concentration range of 1 fM to 0.1 nM with a LOD of 1 fM. Furthermore, the sensors showed excellent selectivity, good sensitivity, rapid response, and acceptable reproducibility. Saleem and Guler developed an electrochemical biosensor for the detection of paracetamol using PdNPs-decorated CGO-modified GCE [57]. Erden and colleagues developed a novel platform using GO and polyvinyl ferrocene (PVFc) modified SPCE for the immobilization of diamine oxidase (DAOx) and/or monoamine oxidase (MAOx), and utilized it for the biosensing of tyramine [58]. Among the two developed sensors, the DAOx-immobilized sensor exhibited better linear range, better selectivity, and a lower LOD than the MAOx-modified sensor in 0.05 M BR buffer (pH 8.5) containing 0.1 M KCl. However, due to their overall lack of selectivity, the developed sensors could not be used to determine biogenic amines individually without using the standard addition method. Subsequently, the sensors were employed for the determination of tyramine in cheese samples, which showed acceptable recoveries. Gupta and colleagues developed an aminoterephthalic acid-functionalized GO (AFGO) for the immobilization of anti-*E. coli* antibodies and utilized it for the biosensing of *E. coli* [59]. The antibody was immobilized by covalently coupling the free amino groups of AFGO and the antibody via EDC/NHS reaction. The electrocatalytic reaction was carried out in KCl with 10 mM potassium ferri- and ferrocyanide as a redox probe. The developed biosensor has various advantages, such as large surface area, abundant terminal functional groups, excellent conductivity, and porosity, which improved the electrocatalytic performance of the immobilized antibodies. However, the large-scale production of graphene derivatives is highly challenging and involves costly physical and chemical deposition methods. In another study, an electrochemical aptasensor was developed for the selective detection of *Cronobacter sakazakii* (CBSK) using a methylene blue-coupled GO (MGO) modified electrode [60]. The biosensor was fabricated by immobilizing the thiolated aptamer on the clean surface of AuE, and then immersing it in phosphate buffer containing MGO. In the absence of CBSK, the developed sensor showed an excellent electrochemical signal due to the redox properties of methylene blue; however, in the presence of CBSK, the electrochemical signal significantly diminished due to the binding between the aptamer and CBSK. Based on this, an electrochemical aptamer was developed, which showed a wide linear detection range with a LOD of 7 CFU mL^−1^.

Povendano and colleagues developed an electrochemical biosensor for 17β-estradiol using a Lac and rhodium NPs (RhNPs) decorated RGO-modified electrode [61]. RhNPs-decorated RGO was directly prepared via one-pot synthesis. Subsequently, Lac was drop coated and cross-linked via Gld coupling. The electrocatalytic activity of Lac was improved by the synergistic effect of RhNPs and RGO. Furthermore, the developed sensor displayed a wide linear range with a low LOD and good sensitivity for 17β-estradiol detection. In another study, an RGO-, AgNPs-, and ssDNA-modified carbon paste electrode (CPE) was fabricated for the electrochemical biosensing of barium ions [62]. Firstly, an AgNPs-decorated RGO was prepared and mixed with graphite powder to prepare the CPE. Subsequently, ssDNA was deposited onto the AgNPs-RGO-CPE by chronoamperometry in order to obtain the desired biosensor. This biosensor was immersed in different concentrations of barium ions and finally, it was immersed in an electrochemical indicator carmoisine solution. The developed biosensor detected barium ions at a LOD of 45 pM. Halder and colleagues developed a polyethyleneimine (PEI) functionalized redox-active RGO for the immobilization of the enzymes ChOx and GOD, and utilized them for the sensing of cholesterol and glucose [63]. Here the PEI acts as the reducing agent as well as the molecular spacer for RGO, and terminal carboxylic groups of RGO were utilized for the coupling of a ferrocene (Fc) redox mediator via EDC coupling. Subsequently, the enzymes GOD and ChOx were dropped over the as-prepared modified electrodes separately in order to obtain the desired biosensors. The developed biosensors showed excellent electrocatalytic oxidation of cholesterol and glucose in wide concentration ranges at LODs 0.5 µM and 5 µM, respectively. Similarly, GNPs-decorated Fc functionalized RGO was synthesized and utilized for the electrochemical sensing of BPA [64]. In another study, polyoxometalate (POM) deposited poly-IL functionalized RGO was synthesized for the immobilization of GOD, and a glucose biosensor was developed [65]. The developed platform has various advantages, such as large surface area, excellent conductivity, good biocompatibility, and redox activity due to the presence of IL, RGO, and POM in the electrode setup.

#### 2.1.3. Carbon Nanotubes

Carbon nanotubes (CNTs) are 1D cylindrical nanomaterials with a few nanometers of diameter and micrometer scale lengths. Structurally, CNTs just appear as rolled-up sheet(s) of graphene, and they are categorized into single-walled CNTs (SWCNTs) and multi-walled CNTs (MWCNTs). CNTs have various unique physiochemical properties, such as high conductivity, large surface area, good thermal and chemical stability, high tensile strength, ease of functionalization, excellent electrochemical properties, and good biocompatibility. Due to these advantages, CNTs are continuously being used in the fabrication of electrochemical sensors and biosensors [66,67]. However, aggregation of CNTs is one of the major challenges faced by researchers, which can be avoided by incorporating various functional groups by covalent modification, by creating defects, or by physically adsorbing other nanostructures over CNTs. Moreover, modification of CNTs by the aforementioned methods improves the conductivity and stability, and imparts specificity to CNTs. An electrochemical biosensor was developed using cytochrome c (Cyt-c) immobilized over MWCNTs-modified SPCE for the biosensing of fentanyl [68]. The Cyt-c enzyme was immobilized over MWCNT/SPCE via Gld cross-coupling and the developed sensor selectively detected fentanyl at a LOD of 86 ng/mL in phosphate buffer (pH 7.5). Moreover, the developed biosensor was extended for the detection of fentanyl spiked in urine samples. In another study, a glucose biosensor was fabricated using in situ grown CNTs over a gold microelectrode printed on a glass substrate [69]. Finally, GOD was encapsulated by a polymer matrix made of paraphenylenediamine (p-PDA) over the CNT/AuE to obtain the desired biosensor. The developed sensor showed excellent electrochemical oxidation of glucose with a LOD of 0.2 µM in phosphate buffer (pH 6.5). Alvarez-Paguay and colleagues developed an H_2_O_2_ biosensor using HRP and hydroxyapatite-functionalized CNTs (HAp-CNTs) modified GCE [70]. HAp-CNT protects the bioactivity and improves the conductivity of immobilized HRP. The developed sensor exhibited a wide linear detection range with a LOD of 1.91 µM for H_2_O_2_-sensing in 0.1 M phosphate buffer (pH 7.0). Subsequently, the sensor was employed for the detection of H_2_O_2_ in real samples, such as fresh and pasteurized milk samples, which showed acceptable recoveries. In a recent attempt, Bravo and colleagues developed a BPA biosensor using MWCNT and Laccase (Lac) bioconjugate [71]. A bioconjugate consisting of Lac, CS, and BMIM TFB ionic liquid in a proportion of 5:5:2 was fabricated in order to improve the stability and electrocatalytic activity of Lac, while MWCNT was used to enhance the electrochemically active surface area and electron transferability of the developed sensor. The developed sensor exhibited a broad linear range with a LOD of 8.4 nM in 0.04 M BR buffer (pH 5.0) and was also extended for the determination of BPA in river water samples. Yang and colleagues developed a point-of-care uric acid-monitoring electrochemical biosensor using urease-modified super-aligned CNTs (SACNT) [72]. Initially, the urease enzyme was uniformly dispersed with CS and amine-functionalized CNTs, which were then drop cased over the SACNT surface, and then Gld was drop casted and incubated at RT for 1 h to complete the cross-linking process. SACNT was incorporated due to its high conductivity, huge surface area, excellent electrocatalytic area, and large contact area for the analytes to interact. In another attempt, a dopamine (DA) biosensor was developed using silver nanoparticle-decorated FMWCNTs-modified GCE, which showed a LOD of 0.277 µM in 0.01 M phosphate buffer [73]. Pal and Majumder developed a label-free biosensor for the ultra-low detection of silver ions using an ssDNA-functionalized MWCNTs-modified AuE via cytosine–cytosine mismatch chemistry [74]. Initially, the AuE surface was modified with D-cysteine via thiol-gold bonding, and then ethylenediamine was anchored via EDC/NHS coupling. Finally, ssDNA-MWCNT was drop casted and incubated for 30 min to obtain the desired biosensor. The developed sensor detected Ag^+^ ions in a wide concentration range with a LOD of 1 pM. Subsequently, the sensor was employed for the detection of Ag^+^ ions in various water samples with spiked concentrations of Ag^+^ ions and it showed acceptable recoveries.

### 2.2. Metal Nanomaterials

Metal nanomaterials (MNMs) of different shapes, sizes, and dimensions, such as nanoparticles, nanosheets, nanocomposites, nanoflakes, and nanodots are being continuously utilized and explored in the design and fabrication of electrochemical biosensors due to the various following reasons: (i) MNMs have a large surface area due to their nano size, which would facilitate better loading of biomolecules on the electrode surface; (ii) since MNMs are made of metal atoms, they have a large number of free electrons, which would facilitate the electron transferability, thereby improving the conductivity and performance of the developed sensors; (iii) many MNMs have excellent biocompatibility, which is a heavily favored factor that keeps the biomolecules active and prevents them from denaturing; (iv) by suitably tuning the shape, size, and functionality of MNMs, researchers can improve the sensitivity and selectivity of the developed sensors; (v) MNMs have excellent catalytic and electrocatalytic properties; and (vi) many MNMs can be synthesized by green methods, thereby reducing environmental hazards and decreasing the overall cost of biosensor development [75,76].

#### 2.2.1. Metal Nanoparticles

Modifying the electrode surface with metal nanoparticles (MNPs) enhances biomolecule loading and improves the electrocatalytic properties of the developed biosensors. A mycobacterium tuberculosis DNA (MTb) biosensor was developed using a simple copper NP (CuNPs) with an ssDNA-modified platinum electrode (PtE) [77]. Here, the CuNPs was electrochemically deposited (−0.39 V for 60 s at 80 °C) over a precleaned PtE using copper salt dissolved in green solvent (deep eutectic solvent: a mixture of choline chloride and urea) as the supporting electrolyte. The applied potential, time, and temperature were optimized for better performance. Subsequently, a well-aligned DNA monolayer immobilization was carried out by immersing the CuNPs/PtE in an ssDNA solution for 5 h and then in mercaptohexanol (MCH). The developed sensor showed a LOD of 1 nM with a sensitivity of 42.5 µA nM^−1^ cm^−2^. Deposition of CuNPs on PtE synergistically enhanced the electrocatalytic properties of ssDNA and a biocompatible microenvironment was provided by the CuNPs, which retained the nativity of the DNA. In another work, a label-free biosensor was developed for COVID-19 antibody tests using citrate-capped spherical GNPs modified with chemically synthesized peptide receptors [78]. The biosensor was fabricated by modifying the GCE surface with a well-dispersed solution of GNPs, and then the peptide layer containing PEP2003 (five amino acids: Asn-Asn-Ala-Thr-Asn-COOH) and S protein were dropped and incubated. Here, purposefully negatively charged GNPs were used in order to firmly adsorb the peptide on the NPs and to prevent their agglomeration, as well as to retain the dispersity of the synthesized GNPs. The developed biosensor is a label-free, fast, accurate, straightforward, and cost-effective method that can be used as a promising platform for the detection of COVID-19. The functionalization of MNPs is an emerging methodology that improves selectivity and inherits the task-specific properties from MNPs. Flavin adenine dinucleotide (FAD) functionalized GNPs were synthesized and utilized as sensing probes for the selective sensing of DA at nanomolar levels (LOD, 525 nM) [79]. Briefly, a gold substrate was self-assembled with a monolayer of mercaptoundecanoic acid (MDA), then covered with a layer of cationic poly(ethyleneimine), and finally, the negatively charged FAD-GNPs were immobilized via electrostatic interaction in order to obtain the desired biosensor. The developed sensor was extended for the detection of DA in human urine samples. Recently, an alkane thiolate-protected gold nanocluster (APGNC, Au_225_(C6)_75_) was synthesized and doped in a xerogel ((3-mercaptopropyl)trimethoxy silane, MPTMS), and embedded with GOD-modified PtE for the biosensing of glucose [80]. Incorporating APGNC into the sensor setup improved the biosensor’s performance in terms of linear range doubling and enhanced sensitivity and selectivity, as well as amplifying the response time up to fourfold, in contrast to the sensor developed without APGNC. The xerogel layer thickness and carbon chain length in APGNC were optimized, as they play a significant role in the performance of the developed biosensor. Similarly, drying time, homogeneity of the xerogel, humidity, and composition of the material on the electrode defines the stability and selectivity of the biosensor. Moreover, the presence of xerogel stabilizes the GOD, whereas the APGNCs improve the electron transferability and electrocatalytic activity of GOD, thereby improving the overall performance of the proposed biosensor. Recently, a urea biosensor was developed by covalently immobilizing the urease enzyme on different silanized ferrite NPs (Cu, Co, Ni and Zn) modified GCE [81]. Here, silanization was performed in order to impart amine functionalization as well as to obtain stable NPs of uniform size and shape. Compared to various ferrite NPs, CuF was shown to give better sensitivity and a low limit of detection for the sensing of urea. The superior performance of CuF over other ferrite NPs could be due to better conductivity and a relatively narrow band gap, which facilitated the electron transfer process. Furthermore, real sample analysis was performed with soil and milk samples using the proposed biosensor, and showed acceptable recoveries.

#### 2.2.2. Metal Oxide and Sulfide Nanoparticles

An electrochemical biosensor was developed by immobilizing GOD on zinc oxide NPs (ZnONPs) and a soy protein film (SPF) modified electrode and utilized for glucose biosensing [82]. ZnONPs offered good conductivity, a high isoelectric point, and electrochemical activity; on the other hand, SPF was chosen as the support matrix for the covalent immobilization of GOD due to its good biocompatibility and stability. Yin and colleagues developed cobalt oxide NPs (CONPs, Co_3_O_4_) decorated N-doped electrospun CNF (carbon nanofiber) for the detection of DA secreted by PC12 cells [83]. The CNF was synthesized by electrospinning, followed by hydrothermal surface functionalization, and the CONPs were electrodeposited on the as-prepared CNF-modified electrode. The developed biosensor had many advantages, such as large surface area, excellent conductivity, good selectivity, and sensitivity towards DA, and exhibited a LOD of 9 nM in 0.1 M phosphate buffer (pH 7.4). Subsequently, it was extended for the detection of DA secreted by PC12 cells. Similarly, a non-enzymatic glucose sensor was developed using a straw sheaf-like CONPs-modified electrode, which showed a high sensitivity of 669 µA mM^−1^ cm^2^ with a LOD of 0.31 µM [84]. Centane and colleagues developed an electrochemical aptasensor by immobilizing the aptamer on cobalt phthalocyanines—cerium oxide (CeO) NP conjugate for the sensing of human epidermal growth factor receptor 2 (HER) [85]. Here, the aptamer HB5 was covalently linked to the modified electrode using DCC/NHS coupling via amide bond formation between the free amine groups of HB5 and free carboxylic acid groups of the conjugate. Furthermore, the developed sensor showed high sensitivity, a low detection limit (0.2 ng mL^−1^), good stability, and improved electrocatalytic activity due to the synergistic behavior between the metal phthalocyanines and metal oxide NPs. In a recent attempt, three-dimensional porous bimetallic oxide NS (BMONS) were developed and utilized for the immobilization of acetylcholinesterase (ACLE) and detection of pesticides omethoate (OME) and dipterex (DPX) [86]. The BMONS were made in combination with different ratios of cobalt and Ni, Cu, or Fe. The sensor was developed by modifying the GCE surface with BMONS and then covered with a layer of graphdiyne (GDY) to facilitate ACLE immobilization by providing a biocompatible microenvironment. BMONS improved the electrocatalytic activity of the ACLE, while GDY preserved the nativity of the ACLE. The proposed sensor showed enhanced electrocatalytic activity and high sensitivity towards pesticide detection and exhibited LODs of 0.033 and 0.36 pM for OME and DPX, respectively in 0.1 M phosphate buffer (pH 7.6). Subsequently, the sensor was employed for the determination of DPX spiked in tap and lake water samples.

**Figure 2:**
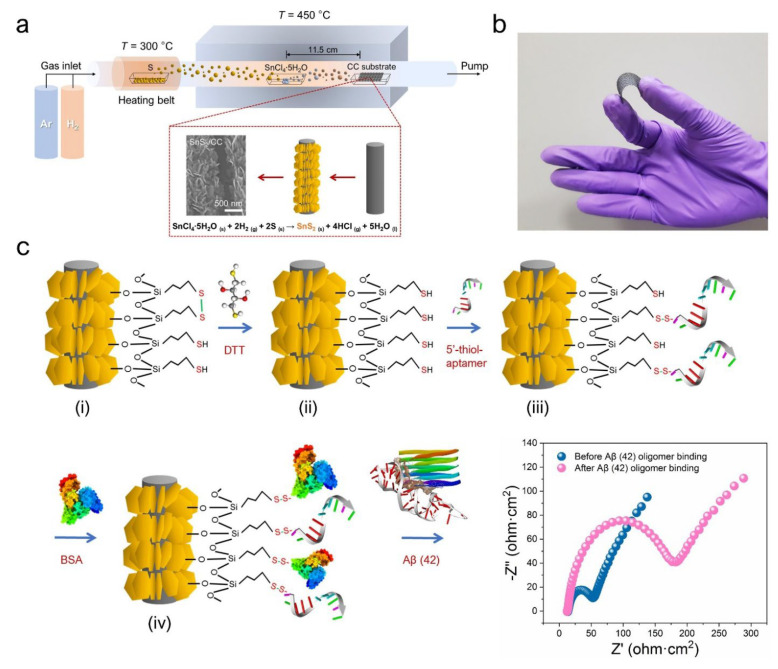
(**a**) Synthesis of tin disulfide NSs on CC, (**b**) flexibility of CC substrate, (**c**) stepwise fabrication of the proposed aptasensor and its electrochemical response. Reproduced with permission from [87].

**Figure 3:**
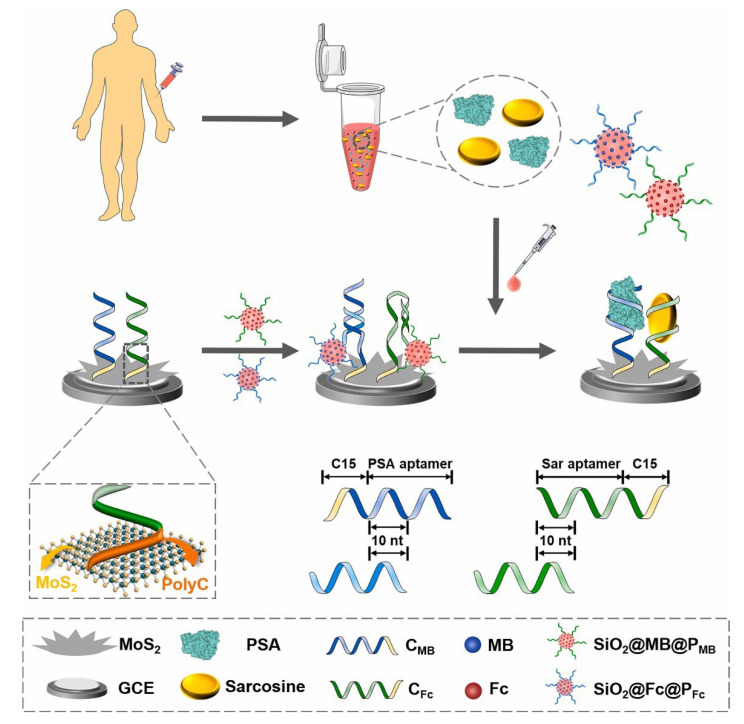
Various steps involved in the fabrication of MoS_2_-based electrochemical aptasensor for the sensing of sarcosine and PSA. Reproduced with permission from [90].

Recently, an ultrasensitive electrochemical aptasensor was constructed using Aβ42 capture aptamer covalently modified over vertically aligned tin disulfide (SnDS) NSs and utilized for Alzheimer’s disease diagnosis [87]. Firstly, SnDS NSs were grown on a carbon cloth (CC) substrate via chemical vapor deposition (CVD) and then functionalized with MPTMS to have terminal thiol groups (Figure 2). Later, the thiol-functionalized Aβ42 aptamer was covalently bonded to the modified CC via disulfide bond formation in order to obtain the desired aptasensor. The constructed sensor displayed excellent stability, good selectivity, and high catalytic activity due to the presence of a large number of edge-plane-active sites as a result of the vertical aligning of SnDS NSs. Moreover, the aptamer was extended for the detection of AβOs in blood serum samples. In another study, Li and colleagues constructed a GOD-immobilized over cobalt sulfide NPs (CSNPs) decorated MWCNT modified GCE and employed it for the realization of direct electron transfer (DET) of GOD and glucose biosensing [88]. The developed sensor had excellent conductivity, good biocompatibility, and a large surface area due to the presence of CSNPs along with MWCNT, which favored high loading of GOD and preserved its bioactivity. Subsequently, DET of GOD was successfully achieved on the newly developed platform, and the lowest detection limit was found to be 5 µM in 0.1 M phosphate buffer (pH 6.0). Moreover, the developed sensor was utilized for the determination of glucose spiked in human serum samples. Rohaizad et al. developed a niobium-doped titanium disulfide (Nb-TDS) for the immobilization of GOD and fabricated a glucose biosensor [89]. Nb-TDS was synthesized separately with different concentrations of Nb and its performances towards glucose sensing were compared. TDS (group 4) was chosen due to its high conductivity, cost-effectiveness, and good stability while being a group 5 element, Nb was doped to improve the electrochemical properties of TDS towards sensing. Among different combinations, Ti_0.95_Nb_0.05_S_2_ showed the best performance, detecting glucose selectively at +0.1 V with a LOD of 25.7 µM in phosphate buffer (pH 7.2) containing 2 mM FcMeOH. Using a flower-like MoS_2_ and SiO_2_ conjugated with DNA probes and electroactive tags, an aptasensor was developed for the simultaneous detection of the prostate cancer biomarkers sarcosine and prostate specific antigen (PSA) [90]. Here, the functional interface MoS_2_ was incorporated to improve DNA hybridization and accelerate the intermolecular accessibility, while the SiO_2_ nanoprobe amplified electrochemical detection (Figure 3). The fabricated aptasensor detected sarcosine and PSA at LODs 14.4 and 2.5 fg mL^−1^, respectively. In another attempt, MoS_2_ NSs decorated with GNPs and bovine hemoglobin (BHb) were constructed for the selective detection of the pesticide chlorpyrifos (CYF) [91]. The presence of MoS_2_ and GNPs offered good conductivity and a large surface area for BHb immobilization and improved the electrocatalytic activity of BHb towards CYF detection. Subsequently, the sensor was explored for its applicability in real samples such as cabbage and leek samples, which showed acceptable recoveries. Similarly, a nonenzymatic electrochemical sensor was developed using AgNPs-decorated MoS_2_ microflowers and was utilized for glucose sensing in 0.1 M NaOH with potassium ferrocyanide as a redox probe [92]. Jeong and colleagues developed an aerogel made of 3D MoS_2_ with GR (MGA) for the immobilization of GOD and employed it for the biosensing of glucose via DET [93]. MGA offered high conductivity and a large surface area with an interconnected framework, which favored better loading of GOD and improved the electrocatalytic performance towards glucose sensing in phosphate buffer solution.

#### 2.2.3. MXenes

MXenes are layered 2D nanomaterials like graphene, and are made of transition metal carbides, nitrides, and carbonitrides. MXenes are formed by acid or alkali etching of the A layer from their MAX phases. Similar to other NMs, MXenes also possess excellent properties, such as high electrical conductivity, good thermal and chemical stability, large surface area, and so forth. However, MXenes are a step ahead of other NMs in terms of the ease of large-scale synthesis, possessing excellent biocompatibility and presence of abundant surface functionalities as a result of etching, and these functionalities can be altered by suitably choosing the etchant and the etching process, due to which MXenes possesses excellent hydrophilicity [94,95,96]. Furthermore, these terminal functional groups can be sensibly utilized for the immobilization of biomolecules, which enhances the stability and performance of the developed biosensors. An H_2_O_2_ biosensor was developed by encapsulating hemoglobin (Hb) between the sheet-like structure of Ti_3_C_2_ MXenes (TMX) [97]. The direct electrochemistry of Hb was able to be achieved due to the excellent conductivity and good biocompatibility of the TMX, which facilitated the rapid electron transfer between the deeply buried heme center of Hb and TMX without altering the nativity of Hb, whereby an effective electrochemical biosensor was developed for H_2_O_2_. In another study, a mediator-free biosensor was developed by immobilizing the TYR enzyme over TMX for the rapid and ultrasensitive detection of phenol [98]. The proposed biosensor has advantages, such as ease of preparation, facilitation of electron transferability, cost-effectiveness, and exhibited superior stability and selectivity for the sensing of phenol. A tetrahedral DNA (tDNA) immobilized TMX was fabricated for the label-free electrochemical detection of gliotoxin [99]. By exploiting the excellent conductivity and large surface area of TMX, tDNA was stably immobilized and utilized for biosensing. The developed sensor exhibited a LOD of 5 pM over a linear detection range from 5 pM to 10 nM for gliotoxin sensing. Subsequently, it was employed for the detection of gliotoxin spiked in human serum samples.

**Figure 4:**
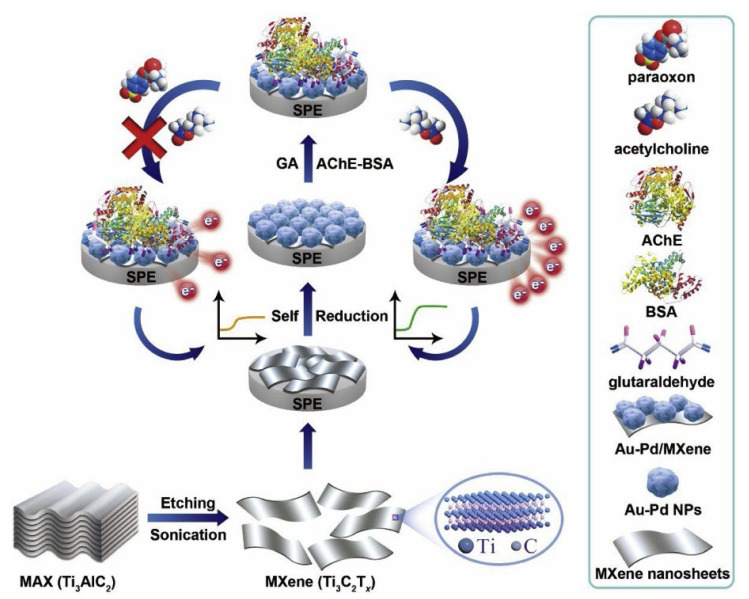
TMX synthesis and biosensor fabrication via covalent immobilization of ACLE. Reproduced with permission from [100].

**Figure 5:**
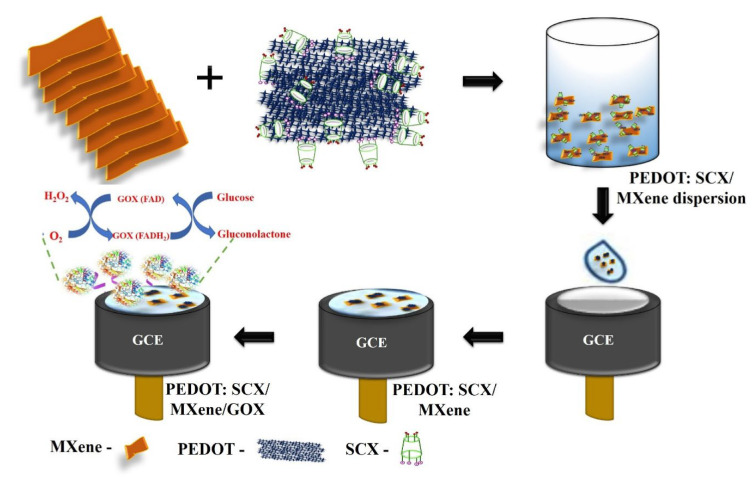
Synthesis of TMX/PEDOT/SCX and stepwise fabrication of glucose biosensor. Reproduced with permission from [103].

The sheet-like structure of MXenes facilitates the deposition/doping of other nanoparticles and materials, thereby synergistically enhancing its own properties. Bimetallic Au–Pd NPs were formed in situ over ultrathin TMX sheet-modified SPE via a self-reduction process and used as a platform for the covalent immobilization of ACLE for the development of a pesticide biosensor [100]. The Au–Pd NPs-modified TMX facilitated better loading of enzymes and improved the electrocatalytic properties of the developed sensor due to its large surface area and excellent conductivity (Figure 4). The fabricated disposable biosensor detected paraoxon at a low concentration of 1.75 ng L^−1^ in 0.01 M phosphate buffer (pH 7.4). Subsequently, real sample analysis was carried out by spiking paraoxon in cucumber and pear samples, which yielded recovery ranges from 87.93% to 111.02%. In another study, a label-free electrochemical biosensor was developed for the detection of microRNA-21 (miR) using TMX modified with MoS_2_, thionine, GNPs, and catalytic hairpin assembly (CHA) [101]. The MoS_2_ NPs were synthesized over TMX nanosheets, and then the MoS/TMX was modified with thionine and GNPs (13 nm) to form GNPs–Thi–MoS/TMX. Finally, the capture probe (CHA) was immobilized over the GNPs to form the desired label-free biosensor. The developed biosensor has various advantages, such as large surface area, improved conductivity, and excellent electrocatalytic activity due to the presence of various nanomaterials such as TMX, MoS_2_, and GNPs on the sensor setup. Moreover, the sensor was able to detect femtomolar concentrations of miR (26 fM) in 0.01 M phosphate buffer, and it exhibited acceptable performances even under clinical conditions; hence, it can be applied to practical applications. Song and team developed an electrochemical pesticide biosensor for methamidophos using microcuboids (MC) derived from MOFs, GNPs-deposited TMX, and an ACLE-modified electrode [102]. Initially, MC was drop casted over the GCE, then modified with GNPs-decorated TMX, and finally covered with ACLE. The developed platform possessed excellent sensitivity, improved conductivity, large surface area, and good biocompatibility towards the immobilized ACLE due to the presence of various NMs in different forms, such as spherical particles, microcuboids, and nanosheets, which synergistically improved the overall performance of the constructed biosensor. Recently, a nanocomposite-based biosensor was developed using GOD immobilized on TMX with poly(3,4-ethylenedioxythiophene) (PEDOT) and 4-sulfocalix [4]arene (SCX) and employed for the glucose biosensing [103]. The DET of GOD was successfully achieved on the proposed biosensor which could be due to the excellent conductivity and biocompatibility of the TMX/PEDOT/SCX nanocomposite (Figure 5). The developed sensor showed a linear detection range from 0.5 to 8 mM with a LOD of 0.02 mM for glucose detection in 0.1 M phosphate buffer. Using TMX and GR, a 3D porous hybrid film was developed for the immobilization of GOD and utilized for the biosensing of glucose [104]. The fabricated platform has excellent porosity, a huge surface area, and a large number of hydrophilic groups as a result of hybrid formation, which favored and stabilized the immobilization of GOD. Moreover, the GOD was well accommodated within the porous network due to its hydrophilic microenvironment and hence, DET was successfully realized on the newly developed hybrid platform.

### 2.3. Quantum Dots

Quantum dots (QDs) are 0D nanostructured semi-conducting materials with a diameter of a few nanometers; in other words, it is merely a collection of a few atoms. QDs have unique optical and electronic properties relative to their bulk and compared to other nanomaterials. Along with the regular properties of nanoparticles, such as large surface area, good electron transferability, and ease of synthesis, QDs possess various other advantages as well, such as high photostability, low cost, excellent synthetic versatility, high quantum yields, and excellent quantum confinement effects. Moreover, the properties of QDs can be tuned by altering their size, shape, composition and structure. For instance, by controlling the size of QDs, one can make the QDs absorb or emit a specific wavelength of light. There are different kinds of QDs, such as carbon-based QDs (including GR, GO, etc.), functionalized QDs, doped QDs, metallic QDs, metal oxide QDs, core-shell QDs, and so forth [105,106,107].

**Figure 6:**
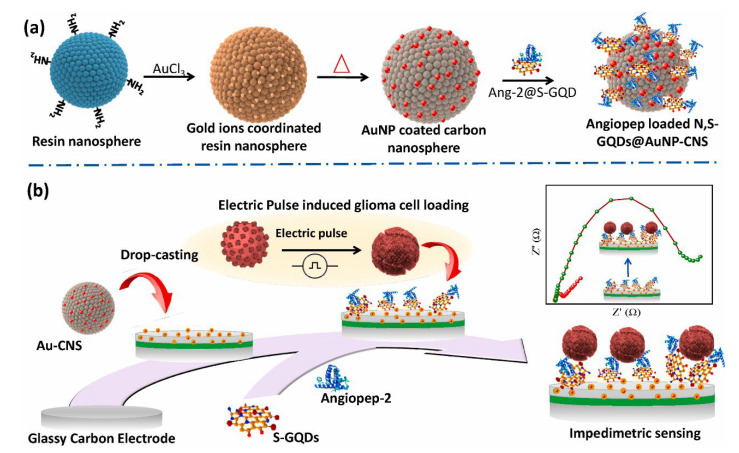
(**a**) Synthesis of angiopep-loaded S-GRQDs/GNP/CNSp, (**b**) Stepwise electrode fabrication and impedimetric detection of glioma cells. Reproduced with permission from [111].

An electrochemical biosensor for epinephrine (EP) was developed using graphene QDs (GRQDs) covalently immobilized with Lac via Gld cross-coupling [108]. A thin layer of GRQDs was formed on the clean surface of GCE, which facilitated the immobilization of Lac due to its large surface area and monodispersed nature. The developed sensor exhibited excellent electrocatalytic oxidation of EP in a wide linear concentration range with a LOD of 83 nM in phosphate–citrate buffer (pH 5.2). However, the developed biosensor showed a feeble interference for ascorbic acid (12%), uric acid (2%), and cysteine (3%) during amperometry measurement. Subsequently, real sample analysis was performed with a pharmacological product Adrenalina WZF samples. Similarly, by using GRQDs, ceramic-coated liposomes (creasome, CRE), and ChOx, an electrochemical biosensor for cholesterol was fabricated [109]. Here, a layer-by-layer strategy was followed for the fabrication. First, CRE was coated over GCE, and then it was immersed in PEI solution to obtain a PEI layer over CRE/GCE; the coating of PEI produces a positive layer over the surface. Subsequently, it was immersed in negatively charged GRQDs. Again, a PEI layer was formed over GRQDs, and then the negatively charged ChOx was electrostatically immobilized to obtain the proposed biosensor. Moreover, GRQDs improved electron transferability between the ChOx and electrode, as well as preserved the bioactivity of the ChOx. The developed biosensor showed excellent electrocatalytic activity towards cholesterol over the concentration range from 16 µM to 6.186 mM with a LOD of 5 µM in phosphate buffer (pH 7.0). In another study, Ye and colleagues developed an electrochemical biosensor for the detection of bacteria response towards antibiotics using amine-functionalized GRQDs covalently immobilized with anti-*Salmonella* antibodies via Gld over a nanoporous alumina membrane [110]. A sulfur-doped GRQDs deposited over a GNPs-decorated carbon nanosphere (CNSp) was developed for the conjugation of angiopep-2 and utilized for the electrochemical detection of glioma cells (glioblastoma multiforme) [111]. S-GRQDs were linked to GNPs via Au–thiol linking, which enhanced the stability and conductivity of the developed platform (Figure 6). The developed biosensor showed a broad linear detection range with a LOD of 40 cells/mL in 0.1 M phosphate buffer (pH 7.5) and was also employed for the detection of glioma cells spiked in human serum samples. Nashruddin and colleagues developed a nanocomposite using PEDOT, polystyrene sulfonate, TMX, and GRQDs for the immobilization of GOD and fabricated a glucose biosensor [112]. Recently, an electrochemical biosensor for miR-141 was developed using GOQDs immobilized with an ssDNA detection probe [113]. The developed biosensor is rapid, label-free, portable and mobile-device-integrable.

An electrochemical biosensor for H_2_O_2,_ released from CA-125, was developed using antimonene QDs (AMQDs) immobilized with catalase (Catl) enzyme [114]. AMQDs were chosen due to their large surface area, good anticancer properties, and excellent biocompatibility, which facilitated Catl immobilization and H_2_O_2_ detection in 0.1 M phosphate buffer (pH 7.5). In another study, a colloidal PbS QDs and gold nanosphere (GNSp) modified electrode was developed for the immobilization of GOD and a glucose biosensor was fabricated [115]. Here, PbS QDs were used due to their large surface area, higher number of active sites, and quantum confinement, while GNSp offered excellent conductivity and good biocompatibility toward biomolecules. A DNA biosensor was constructed for the gender determination of Arowana fish using mercaptopropionic acid (MPA) capped ZnS QDs (MZSQ), GNPs, and cysteamine (cys) [116]. The bare SPE was coated with GNPs, and then cys was allowed to form a thiol linking with GNPs. The coupling of the amine ends of cys with the carboxylic acid groups of MZSQ was performed via EDC/NHS coupling. Later, the other ends of activated carboxylic groups of MZSQ were allowed to bond with amine-terminated probe DNA. The developed DNA biosensor showed an excellent electrochemical response when binding with target DNA. In another attempt, L-cysteine-functionalized ZnS QDs were developed for the fM detection of miR-200a, an ovarian cancer biomarker [117]. Richard and colleagues developed a label-free electrochemical DNA biosensor using a nanocomposite made of SnO_2_ QDs and GNPs for the detection of lung cancer biomarkers [118]. A nanocomposite-based electrochemical biosensor was developed using MPA-capped CdTe/CeSe/ZnSe QDs covalently linked to poly(propylene imine) (PPI) via EDC/NHS coupling and utilized for the immobilization of ChOx and detection of cholesterol [119]. Initially, the PPI was electrodeposited over the GCE, and QDs were attached to it via amide bond formation, and then ChOx was immobilized by immersing the QDs/PPI/GCE in ChOx solution. The fabricated sensor showed a linear range from 0.1 to 10 mM with a LOD of 75 µM. Table 1 displays the electrocatalytic performances of various functional nanomaterials based electrochemical biosensors.

### 2.4. Organic Frameworks

Organic frameworks (OFWs) are a group of compounds that have one-, two-, or three-dimensional ordered crystalline porous structures and are formed by many repeating units joined together to obtain a cage- or frame-like structure. If the framework structure contains metal (mono or multi) clusters/ions linked together with organic (bi- or multifunctional) linkers, then they are called metal organic frameworks (MOFs). On the other hand, if the repeating units are made of lighter elements such as H, B, C, N, O, etc., then they are known as covalent organic frameworks (COFs). In general, MOFs and COFs have many interesting properties, such as large surface area, tunable porosity, moderate to good conductivity, excellent synthetic versatility, and good biocompatibility. Moreover, these properties can be tailor-made to meet the requirements of specific applications. For example, the conductivity and stability of the OFWs can be improved by doping with nanoparticles and other conducting supports. Similarly, the pore size and structural geometry of OFWs can be tailored by strategically choosing the organic linkers, reaction conditions, etc.

#### 2.4.1. Metal Organic Frameworks

MOFs are not new to the field of material sciences; they have been utilized from 1995 onwards. Over the past decade, they have seen a boom, and so far, over 100,000 MOFs have been synthesized, and millions of possible MOF structures are being predicted in silico. The metal clusters are often called nodes, and the functional ligands are known as linkers or spacers [120,121,122]. An electrochemical biosensor for the detection of the pesticide TCA and the food preservative sodium nitrite was developed using GNPs electrodeposited on a magnesium MOF immobilized with Mb [123]. Here, the Mg-MOF was chosen due to its large surface area, high stability, and excellent porosity, which aid in the immobilization and stabilization of Mb. Furthermore, GNPs were incorporated on the Mg-MOF surface via electrodeposition in order to enhance the conductivity and electrocatalytic activity of the fabricated biosensor. The developed sensor electrochemically reduced TCA and nitrite at the reduction potentials of −0.298 V and −0.535 V (vs. Ag/AgCl), respectively, in phosphate buffer (pH 2.0). However, the proposed biosensor showed higher LOD values for TCA than many reported sensors. Subsequently, the developed sensor was employed for the detection of TCA in medical skin lotion samples, which showed recovery values between 102.6% and 104.7%. Similarly, GNPs electrodeposited on a MoS_2_-supported Cu-MOF were developed and utilized for the immobilization of the CA125 antibody and a biosensor for the detection of the ovarian cancer biomarker CA125 was fabricated [124]. MoS_2_ was supported to enhance the electron and ion transferability of Cu-MOFs, and GNPs were incorporated in order to improve the conductivity between the antibody and Cu-MOF. The fabricated sensor demonstrated excellent electrocatalytic activity and good selectivity towards CA125 detection over a concentration range from 0.5 mU mL^−1^ to 500 U mL^−1^. In another study, GNPs-decorated Cu-MOF was developed for the immobilization of a capture probe based on CHA-HCR (hybridization chain reaction) and an enzyme-free biosensor for the detection of MiR was fabricated [125]. Here, the huge surface area and excellent porosity of Cu-MOFs were exploited for the decoration of GNPs and immobilization of the capture probe. The developed Cu-MOF system also served as the signal probe by producing electrochemical signals as a result of capture probe hybridization. A magnetic Fe_3_O_4_@ZIF-8 MOF (MMOF) based nanozyme was developed for the in-situ detection of H_2_O_2_ released from cardiac cells [126]. The nanozyme consists of a base layer of MoS_2_ NSs, which was coated with as synthesized MMOFs and finally covered with a layer of gold nanoflower (GNFs) via electrodeposition. The base MoS_2_ and final GNFs layers were incorporated to improve the conductivity, electrocatalytic activity, and long-term stability of the nanozyme, as well as to retain the surface states and catalytic activity of MMOFs.

Functionalization of MOFs was carried out in order to gain more control over the structure, reactivity, and specificity of the developed MOFs. A folic acid (FA) functionalized Zr-MOF (UiO-66) was developed for the enzyme-free electrochemical detection of cancer cells [127]. Zr-MOF provided a large surface area and good surface roughness; additionally, FA has a good affinity to folate receptors (FR) and easily binds with them. Hence, the developed FA-Zr-MOF was successfully applied for the detection of FR-rich cancer cells and showed the lowest detection limit of 90 cells mL^−1^. In another study, a nucleic acid (NA) functionalized UiO-66 was developed and utilized for the simultaneous determination of tumor biomarkers [128]. By exploiting porosity, the NA-MOF was utilized as a nanocontainer for loading redox dyes, and closed by the gatekeeper dsDNA. As a result of recognition and hybridization, the dsDNA separates from the MOF, and the electroactive redox dyes are released, which produces a measurable signal. The proposed sensor identified the tumor biomarkers MiR and let-7a simultaneously at LODs 8.2 and 3.6 fM, respectively. Subsequently, the sensor was employed for the detection of MiR and let-7a spiked in human serum and serum samples obtained from breast cancer patients, which showed acceptable recoveries. A PdNPs-embedded amine-functionalized Cr-MOF (MIL101) was synthesized and utilized for telomerase activity detection [129]. Similarly, GNPs-decorated Ce-MOF were developed for the ratiometric detection of telomerase activity [130]. Huang et al. developed an amine functionalized Co-MOF decorated with GNPs for the immobilization of Cyt-c-modified MWCNT and fabricated a nitrite biosensor [131]. Here, the Co-MOF provided ample surface area to decorate the GNPs and immobilize Cyt-c/MWCNT. On the other hand, GNPs and MWCNT bestowed biocompatibility, coupled with a conductive microenvironment for the stabilization of Cty-c on the developed platform. The developed biosensor demonstrated excellent electrocatalytic detection of nitrite over a broad linear concentration range with a LOD of 4.4 nM in 0.05 M phosphate buffer (pH 7.4). Subsequently, it was utilized for the determination of nitrite spiked in apple and sausage samples. Gupta and colleagues developed a nanocomposite using Cu-MOF and polyaniline (PANI) for the covalent immobilization of antibodies and fabricated an *E. coli* biosensor [132]. The large surface area and carboxylic functionality of Cu-MOF favored the immobilization of antibodies. In addition, the excellent conductivity and biocompatibility of PANI improved the overall conductivity of the platform and aided in retaining the nativity of the biomolecule. In another study, AgNPs embedded on a poly-Zr-MOF(polyUiO-66) was developed for the immobilization of antibodies and aptamers and fabricated electrochemical biosensors for the detection of H1N1 and SARS-CoV2 [133]. The developed platform has various advantages, such as large porosity, specific surface area, rich functionality, excellent electrocatalytic activity, and biocompatibility due to the presence of poly-Zr-MOF and AgNPs, which improved the electrocatalytic activity of the proposed biosensor.

#### 2.4.2. Covalent Organic Frameworks

**Figure 7:**
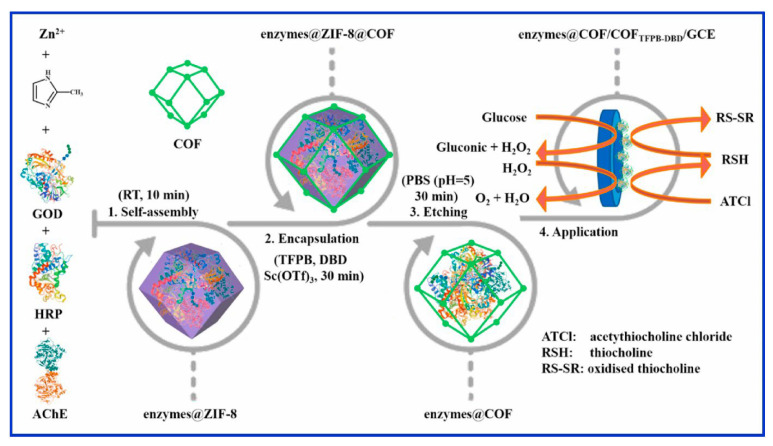
Synthesis of GOD, HRP, and ACLE-loaded COF microcapsules and the electrochemical biosensing of glucose, H_2_O_2_ and malathion. Reproduced with permission from [144].

COFs are porous crystalline organic polymers composed of building blocks of monomer precursors. In other words, a COF is a repeating combination of two or more covalently connected organic molecules in a 2- or 3-dimensional framework. The degree and the intrinsic order are pre-determinable, and they can be rationally designed by suitably choosing the monomer with the desired functionality at the desired position. COFs have superior properties, such as large surface area, good thermal stability, low density, excellent porosity with tunable pore size, synthetic versatility, and so forth [134,135,136,137]. A triazine-based 2D COF (TCOF) was developed for the immobilization of superoxide dismutase (SOD) and an electrochemical biosensor for the biosensing of superoxide anions was fabricated [138]. The biosensor was developed by drop casting the composite (optimized quantities of TCOF, SOD, Gld, and gelatin polymer) over a precleaned SPE. TCOF improved the loading, stability, and activity of SOD due to its excellent porosity, large surface area, good conductivity, and biocompatibility. The developed COF-based biosensor showed a linear detection range from 10 nM to 100 µM with a LOD of 0.5 nM for the detection of superoxide anions. However, to reach the steady state current, the developed sensor took approximately 30 s during the amperometry measurements. Subsequently, the sensor was tested for its clinical applicability by analyzing healthy and cancerous tissue samples, which showed a good response. Xiao and colleagues developed a biosensor for carbaryl detection using nitrogen- and oxygen-rich COFs modified with ACLE [139]. The free terminal groups such as -NH, -OH and C=O of COFs were utilized for the covalent coupling of ACLE within the pores of COFs, thereby stabilizing and improving the electrochemical activity of ACLE. During electrocatalysis, ACLE turns to thiocholine (TCL), which attracts the ferri- and ferrocyanide anions in the electrolyte solution, thereby showing a well-defined redox signal, whereas in the presence of carbaryl, TCL formation was inhibited and hence a poor redox signal was observed. Using this phenomenon, a paper-based turn-off electrochemical biosensor was constructed, which showed a linear detection range from 0.48 to 35 µM with a LOD of 0.16 µM in 0.1 M KCl with 5 mM of potassium ferri- and ferrocyanide as a redox probe. In another attempt, HRP was covalently immobilized over a magnetic COF and an electrochemical biosensor for the determination of hydroquinone (HQ) was developed [140]. The magnetic COF was synthesized in situ by adding Fe_3_O_4_ during COF formation. Thereafter, HRP and Gld were added to the magnetic COF and allowed the HRP and COF to covalently couple. The iron oxide particles were accumulated within the pores of COFs and the HRP molecules covalently bonded to the terminal functional groups of the COF. The developed electrocatalyst was drop coated over GCE and employed for the determination of HQ, which showed a LOD of 120 nM in 0.1 M phosphate buffer. Subsequently, it was employed for the determination of HQ spiked in tap, lake, and wastewater samples, which showed recovery ranges between 98.4% and 104.8%. Similarly, GNPs-deposited magnetic COF was developed for the immobilization of DNA substrate and utilized for the detection of adenosine triphosphate (ATP) [141]. The GNPs were well confined in the uniform nanoporous structure of magnetic COFs, which prevented them from agglomeration. Furthermore, the presence of GNPs improves the electronic conductivity and electrocatalytic activity of the developed electrocatalyst. The developed electrocatalyst successfully reduces 4-nitrophenol (4-NP) under normal circumstances. However, in the presence of ATP, the 4-NP reduction was suppressed due to the formation of dendritic DNA strands. Based on this, an ATP biosensor was developed, which showed a broad linear detection range with a LOD of 1.6 pM and was further extended for ATP detection in serum samples. Han and colleagues developed an exosome biosensor using COF conjugated with DNA and HRP [142]. Here, the pores in the COFs acted as scaffolds: HRPs to amplify the electrochemical signal and DNAs to identify the target exosomes. The developed sensor showed excellent selectivity and sensitivity over the linear range of 10^4^ to 10^7^ particles per µL. Zheng and colleagues developed a PSA biosensor using Au- and Pt-decorated MnO_2_-functionalized COFs [143]. A multienzyme-based biosensor was developed for the sensing of multiple analytes using microcapsules constructed from COF [144]. The COF microcapsules were embedded with GOD, HRP, and ACLE in a capacious microenvironment of around 600 nm-sized shells (Figure 7). The microcapsules protected the enzymes’ bioactivity by covering them in a biocompatible shell, and it simultaneously ensured their structural conformation in order to preserve nativity. The developed biosensor was successfully employed for the detection of glucose, H_2_O_2_, and malathion in the lowest detection limits of 0.85 µM, 2.81 nM and 0.3 pg/mL, respectively, in 0.2 M phosphate buffer (pH 7.0). Even though the proposed work appears interesting, and presents a new concept for the development of multienzyme biosensors, the fabrication methodology is complex and does not guarantee the preservation of the bioactivity of the biomolecules.

### 2.5. Ionic Liquids

Ionic liquids (ILs) are smart molecules, completely made of ions, such as cations and anions, and held together by the electrostatic force of interactions. Unlike other molecules, ILs are entirely composed of charged species nothing but positive and negative ions and thus, it possesses various unique physiochemical properties, such as high conductivity, larger electrochemical stability, good biocompatibility, excellent thermal stability, less toxicity, non-volatility, negligible vapor pressure, and excellent synthetic versatility [145,146]. Another interesting advantage is that these physiochemical properties can be altered and tuned, depending upon the intended applications, such as changing the solubility from hydrophilic to hydrophobic, tuning the viscosity from less viscous to more viscous, or changing the physical state from solid powder to liquid gel, and suitable functionality can be introduced either on the cation or anion, or even on both the ions. Since, ILs interact with biomolecules in a non-destructive way, they are favorable candidates for the immobilization and stabilization of biomolecules such as enzymes, proteins, DNA, antibodies, etc. Due to these reasons, ILs are being considered as a potential candidate for the fabrication of electrochemical sensors and biosensors [147,148]. In the beginning, ILs were only used as conducting binders in the fabrication of composite electrodes, such as CPE in electrochemical sensors. However, after the exploration of IL possibilities in electrocatalysis, researchers have started using ILs as electrode modifiers and that is how IL-modified electrodes came into the fabrication of electrochemical sensors. Furthermore, due to their excellent biocompatibility and enhanced conductivity, ILs are employed in the development of electrochemical biosensors in order to preserve nativity, as well as to improve the activity of the immobilized biomolecules [149,150].

#### 2.5.1. Functionalized ILs

**Figure 8:**
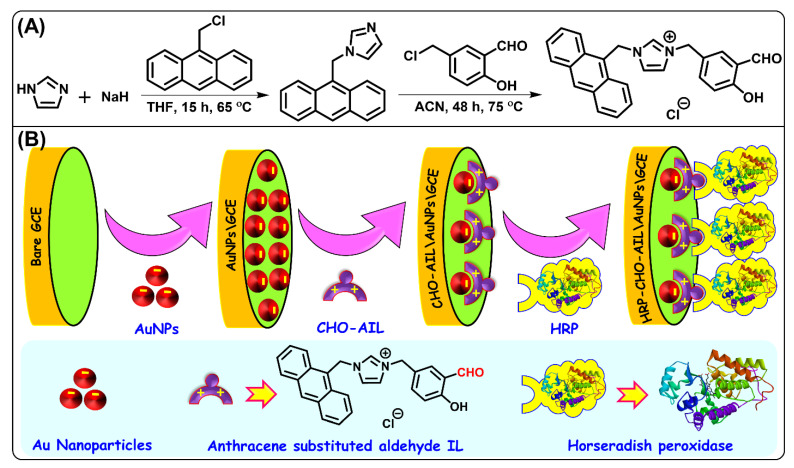
(**A**) Scheme for the synthesis of redox-tethered aldehyde-functionalized IL, (**B**) various steps involved in biosensor fabrication. Reproduced with permission from [153].

A novel amine-functionalized IL (AMIL) was rationally synthesized for the covalent immobilization of Hb and employed for the biosensing of bromate [151]. Initially, the GCE was drop coated with AMIL, and then coated with terephthaloyl chloride (TPC), a bifunctional linker, in order to form a stable amide bond between the AMIL and TPC. Later, an optimized volume of Hb was drop casted over the TPC-AMIL and allowed for covalent immobilization with the other terminal of TPC to form Hb-AMIL/GCE. The direct electrochemistry of Hb was successfully achieved on the newly developed biosensor platform, which showed a wide linear detection range with a LOD of 3 µM for the detection of bromate in nitrogen-saturated 0.1 M phosphate buffer (pH 7.0). Subsequently, in order to simplify the sensor setup, an aldehyde functionalized IL (AFIL) was synthesized for the direct covalent immobilization of GOD without any usage of a bifunctional linker, and a glucose biosensor was fabricated [152]. The biosensor was constructed by drop casting the GO over a SPCE and electrochemically reducing it by potential cycling to obtain ERGO/SPCE. Later, AFIL was dropped to form AFIL/ERGO/SPCE, and then immersed in the GOD solution to covalently immobilize the GOD to AFIL via Schiff base condensation (GOD-AFIL/ERGO/SPCE). The fabricated biosensor was utilized for the electrochemical biosensing of glucose, which showed a wide linear detection range with a sensitivity and LOD of 17.7 µA/mM/cm^2^ and 17 µM, respectively, in 0.1 M phosphate buffer (pH 7.0). As an extension of the previous work, a redox group was tethered to the AFIL (RTAFIL) and developed a nanoconjugate with GNPs for the covalent linking of HRP via Schiff base condensation, which was utilized for H_2_O_2_ biosensing [153]. To fabricate the biosensor, the pre-cleaned GCE was initially modified with negatively charged citrate-capped GNPs, to which bulky positive RTAFIL was immobilized and enabled it to form a nanoconjugate through electrostatic interaction. Finally, the HRP was covalently immobilized over RTAFIL/GNPs/GCE via imine bond formation between the -CHO group of RTAFIL and -NH_2_ group of HRP, as shown in Figure 8. The obtained biosensor showed a LOD of 3.7 µM in 0.1 M phosphate buffer. Moreover, the developed sensors showed excellent sensitivity and selectivity towards the target analytes, and exhibited remarkable stability with acceptable reproducibility. The excellent performance of the developed sensors was due to the covalent immobilization of these enzymes with ILs, which enhanced the electrocatalytic activity and performance of the developed biosensors.

Zappi and team developed a novel platform for the immobilization of Lac (Trametes Versicolor) using cholinium amino-acid-based IL (CAA-IL) through Gld cross-coupling, utilized for the biosensing of gallic acid (GA) [154]. Initially, the carboxylic acid-functionalized MWCNTs (C-MWCNT) and CAA-IL were dispersed to form a uniform suspension, to which Lac and Gld were added and completion of the cross-linking process was allowed. The obtained composite was immobilized over a GCE and allowed to dry in order to obtain the desired biosensor. The developed biosensor was effectively utilized for the determination of GA, which showed a broad linear concentration range with a LOD of 3 µM. Subsequently, the biosensor was extended for the detection of polyphenolic compounds in real samples, such as wine and tea products, which showed recovery values in the range of 90 to 98%. The proposed platform possesses various advantages, such as good electron transfer efficiency, acceptable stability, and excellent biocompatibility due to the presence of MWCNTs along with CAA-IL, which preserved Lac nativity and enhanced its activity. Recently, Yao and team developed a novel soft matrix using aspartic acid (ASA) based imidazole IL (ASAIM-IL) for the stable immobilization of GOD, and utilized it for the biosensing of glucose [155]. The sensor was constructed by drop casting the previously mixed ASAIM-IL/GOD on the GCE surface and allowing it to dry at 4 °C; it was then covered with a layer of Nf solution. The developed sensor showed a linear detection range from 1 µM to 12 mM with a LOD of 0.572 µM and exhibited excellent selectivity and reproducibility. Furthermore, the proposed sensor was employed for the determination of glucose in human serum samples, and compared with the reference method enzyme catalytic spectrophotometry, which yielded a relative error of <5%. The superior performance of these methods could be due to the inclusion of AA and IL into the electrode platform, which preserved the biomolecules’ nativity, enhanced their electrocatalytic activity, and improved the overall performance of the developed biosensors.

#### 2.5.2. ILs with Nanomaterials

A molecularly imprinted polymer (MIP) biosensor was developed for BSA using CS, IL, and GR nanocomposite-modified GCE [156]. Briefly, the GCE was drop casted with IL-GR dispersion and allowed to dry under IR lamp, and then the CS solution (4% *w*/*v* was drop casted in order to obtain CS/IL-GR/GCE (mGCE). Thereafter, the electrode was subjected to electropolymerization by performing potential cycling in the presence of BSA and pyrrole to form the BSA@MIP/mGCE. Finally, the prepared electrode was immersed in 1 M H_2_SO_4_ in order to remove the BSA from the template and to form the MIP-based sensor. The developed MIP sensor exhibited a linear detection range of 0.1 ng/L to 100 µg/L with a LOD of 0.02 ng/L in phosphate buffer containing 0.1 mM of potassium ferri- and ferrocyanide (pH 7.0). Moreover, the sensor displayed excellent selectivity and stability with acceptable reproducibility, and was utilized for the detection of BSA in bovine plasma samples, which displayed recoveries from 97.0% to 101.6%. In another study, a paper-based electrochemical biosensor for glucose was developed using a screen-printed IL (MPPy TFSI) and GR electrode (SILGE) modified with Prussian blue (PB), MXene (Ti_3_C_2_T_x_) nanocomposite and GOD [157]. At first, IL-GR paste was used to print the WE and CE on the paper substrate and the WE was further modified with PB-MXene composite (PB/TMX/SILGE). Finally, GOD was drop casted and covered with a layer of Nf to obtain the paper-based screen-printed biosensor, which was able to sense glucose in a wide range of concentrations with a LOD of 24.5 µM. Subsequently, the developed sensor was employed for the detection of glucose in blood plasma samples, and it exhibited an acceptable correlation with the reference method hexokinase. Moreover, the proposed method opens a new path for the design and development of paper-based printed sensors.

A composite platform based on IL (AMIM TFSI) and GO was developed for the immobilization of choline oxidase (CLO) and ACLE, employed for the electrochemical biosensing of choline (CL) and acetylcholine (ACL), respectively [158]. The biosensor was developed by immersing the precleaned GCE in a solution containing a 1:1 ratio of IL and GO and then allowing it to dry under a slow current of hot air, to which CLO was dropped and incubated at 4 °C for 6 h (CLO/IL-GO/GCE). Separately, ACLE/IL-GO/GCE was fabricated by following a similar procedure and ACLE was used in place of CLO. The developed biosensors displayed a linear detection range from 5 to 1000 nM with LODs of 1.352 and 0.885 nM for ACL and CL, respectively in phosphate buffer (pH 7.4). The proposed platform has advantages, such as increased enzyme loading, enhanced conductivity, and good biocompatibility due to the presence of GO and IL in the sensor setup. Subsequently, it was employed for the determination of ACL and CL in human serum samples. In another study, Isin and colleagues developed an electrochemical biosensor using a GO and IL (BMIM HFP) modified pencil graphite electrode (PGE) for the detection of the breast cancer 1 gene (BRC) [159]. Initially, the PGE (electrochemically activated) surface was modified by dipping it in the IL-GO solution (7.5% IL) for 15 min., then dried in an open atmosphere to obtain IL-GO/PGE. The obtained electrode was immersed in the previously hybridized solution containing the BRC probe and target to obtain the desired biosensor via peptide bond formation between the amino groups of hybrids and carboxylic groups of GO. The successful immobilization of the DNA probe was confirmed by performing electrochemical impedance spectroscopy (EIS), which showed the largest charge transfer resistance (R_ct_) for the final electrode, compared to the former electrodes. Moreover, the developed biosensor is simple, cost-effective, and has good stability and excellent selectivity among other complementary target sequences.

Fan and colleagues developed a dual-enzymatic sensor using IL-MWCNT with Catl and GOD-modified electrodes for the sensing of glucose in food samples [160]. Initially, optimized quantities of IL (BMIM HFP), C-MWCNT, and CS were mixed and yielded a uniform suspension, which was then drop casted over GCE to form IL/C-MWCNT/CS. Thereafter, Catl, GOD, and CS were drop casted one after the other over the modified electrode and allowed to dry at 4 °C to obtain the desired biosensor. Before development, the authors optimized the loading of enzymes and drying time with different ILs, and used the best combination in order to fabricate this biosensor. The developed biosensor showed excellent stability, high sensitivity, and acceptable reproducibility towards glucose sensing. Subsequently, the developed sensor was employed for the determination of glucose in food samples such as milk tea, tomato sauce, drink powders, chrysanthemum crystals, and fruit beverages, which showed good agreement with the enzyme colorimetric method. The superior performance of the developed sensor is due to the synergetic effect of Catl and GOD along with the presence of IL-MWCNT in the sensor platform. In another study, a topotecan (Tpn) biosensor was developed using DNA-modified CPE fabricated using GQDs and IL [161]. The biosensor was constructed by filling the copper-wire-fitted glass tube with previously mixed optimized quantities of GQDs and IL (BP HPF) paste, and the ds-DNA immobilization was performed by immersing the GQD/IL CPE in ds-DNA-containing acetate buffer solution and applying a fixed potential of +0.5 V to for 250 s. Tpn accumulation on the sensor was achieved by dipping the sensor in different concentrations of Tpn and applying an open circuit potential. The developed DNA biosensor is cost-effective, simple, rapid, and selective, as well as sensitive towards the target Tpn. Moreover, it was extended for the determination of Tpn spiked in human serum and urine samples, which showed recoveries from 97.3% to 104%. Table 2 displays the electrocatalytic performances of various functional molecules based electrochemical biosensors.

## 3. Summary and Future Perspectives

There are many non-curable diseases and hazardous chemical pollutants around the world, and they account for millions of deaths every year. Moreover, identifying these diseases in their early stages might give us a chance to eliminate these diseases, or could suppress the disease growth rate. Therefore, it is indispensable to detect and quantify various analytes from biofluids and environmental samples [162,163,164,165]. Electrochemical sensors and biosensors play a significant role in the detection and quantification of biologically and environmentally important analytes due to their high selectivity and sensitivity towards target analytes, and even a small change in concentration would show an immediate response. Furthermore, electrochemical biosensors are robust, cost-effective, offer rapid analysis, require less sample volume, and display excellent disposability and ease of analysis. Moreover, they can be miniaturized into portable handheld devices, which increases their applicability in real-time monitoring and on-site detection [166,167,168]. Thus, enormous efforts have been dedicated towards the design and development of novel electrochemical biosensors, due to which significant advances have recently taken place in the field of electrochemical biosensing, aptasensing, and immunosensing. Furthermore, electrochemical-based technologies such as wearables, continuous monitoring devices, implants, chips, and microfluidic devices are being developed [169,170,171,172,173,174]. Thus, we sought to write a summarized review of the recent advancements in the design and fabrication of electrochemical biosensor platforms using various tailor-made functional materials, molecules, and their sensible hybrids. This review is broadly classified into carbon and metal nanomaterials (particles, sheets, and dots); functional materials and molecules (frameworks and ionic liquids). Furthermore, it elaborates on the significance of the rational design of platform materials and their effects on electrocatalytic activity and biosensing performance. Even though electrochemical biosensors have existed for decades and have seen significant advances on a laboratory scale, only a small number of commercial products are available on the market. There are many challenges ahead that need to be addressed in order to produce successful commercial products.

Rational design of the sensor platform, followed by the sensible immobilization of biomolecules, are the crucial components in the fabrication of effectual electrochemical biosensors. The biomolecules are highly sensitive, and even a slight change in the microenvironment might result in denaturation and loss of biosensor performance [175,176]. The following are some of the key points that need to be kept in mind while designing biosensor platforms. The intended platform or the materials chosen for the fabrication of the biosensor platform should be biocompatible and nontoxic to biomolecules, since the biomolecules are made of polypeptide layers and the secondary and tertiary structures are held together by weak forces, such as hydrogen bonding, electrostatic, disulfide, and Van der Waals forces, which may unfold and disintegrate if the biocompatibility is not maintained. The second crucial factor is conductivity; the intended platform material should be highly conductive, since the active centers are deeply buried inside the thick, non-conducting polypeptide layers, and the material should be capable enough to connect to or communicate with the active centers. For instance, the FAD redox center in GOD, the iron center in Hb, and Cyt-c are all deeply buried inside the protein layers and in order to realize its activity, the platform should communicate the active centers without damaging the secondary and tertiary structures [177,178]. The third important factor is the method of coating or immobilization of the biomolecules on the modified electrode surface. Some of the commonly used strategies are physical mixing or composite formation; drop casting (adsorption); dip coating; entrapping or encapsulating within the pores, cavities, or covers with polymer membranes such as Nafion, etc.; covalent bonding, cross-coupling, SAM and so forth. If the biomolecules are physically held on the surface by adsorption or drop casting, there is a possibility that the biomolecules may leach out during potential cycling. On the other hand, entrapment or encapsulation of biomolecules within the porous network may retain the biomolecules more effectively than adsorption and physical mixing, but they may not involve actively, due to the improper orientation of biomolecules. Therefore, the orientation of the immobilized biomolecules also needs to be maintained in order to display an effective performance. Covalent binding via a bifunctional linker and SAM formation directed by specific functional groups may produce more stable and effective biosensors, since the biomolecules are coupled to the platform strongly and uniform orientations are maintained properly. Moreover, direct covalent immobilization is preferred compared to other methods, since it does not require a bifunctional linker or other external components, such as polymers or Nafion, in order to hold the biomolecules on the substrate. Furthermore, the stability of the fabricated platform plays a significant role in the performance of the developed biosensor, and its stability depends on how well the enzymes are immobilized on the biosensor platform. Therefore, a stable and effective immobilization of biomolecules improves the shelf life and performance of fabricated biosensors. Moreover, the developed platform should have a large surface area, so that more biomolecules could be accommodated on its surface. Finally, the reaction and storage conditions also matter and determine the stability and functioning of the biosensor. For instance, if the pH of the electrolyte solution is too low or too high, it might inhibit the biomolecules’ activity and result in poor performance. Similarly, if the temperature and/or the electrochemical reaction potential is too high, it would denature the biomolecule permanently and result in the failure of the biosensor. Therefore, in order to fabricate effectual biosensors, it is necessary to be prudent and pay attention to the aforementioned requisites.

Among the various materials and molecules elaborated upon in this review, nanomaterials-based biosensor platforms offer high conductivity, large surface area, and excellent electron transferability to the immobilized biomolecules, yet they suffer from potential issues with biocompatibility and stability. Over a period of time, the immobilized biomolecules would denature and leach out from the electrode surface, leading to poor performances. Conversely, functional molecule-based platforms offer excellent biocompatibility and favor the proper orientation of the immobilized biomolecules, yet they possess less conductivity and reduced electrocatalytic activity. These drawbacks have been suppressed by the development of hybrid systems which have functional molecules that facilitate uncomplicated or direct anchoring of biomolecules and conductive nanomaterials to enhance the electron transferability and catalytic activity of the fabricated biosensors. Therefore, in order to construct an effectual biosensor, one should be familiar with the choice of electrode-modifying materials and biomolecule immobilization methodology. Moreover, this review opens a new avenue in the judicious fine-tuning of materials and molecules for the fabrication of multifunctional biosensor platforms, which lays the path for the construction of wearable, implantable, multianalyte sensing devices.

## Figures and Tables

**Table 1:** Electrocatalytic performance of various nanomaterials based electrochemical biosensors.

Electrode	Analyte	Technique	Linear Range	LOD	Sensitivity	Ref.
GCE/fCB/DHDP/TYR	Catechol	CA	1–39 µM	87 nM	539 mA/M	[42]
GCE/CB/CGO/Phage EP01	*E. coli*	EIS	10^2^–10^7^ CFU/mL	11.8 CFU/mL	-	[43]
CB/Conductive C/GOD/Nf	Glucose	CA	50 µM–2 mM	-	-	[44]
GCE/GR/GNPs/ChOx	H_2_O_2_	CA	10 µM–14 mM	25 nM	124.57 µA/µM/cm^2^	[51]
GCE/GR/GNPs/ChEs	Cholesterol	CA	25 µM–0.35 mM	50 nM	3.14 µA/µM/cm^2^	
GCE/GR/GNPs/TYR/CS	BPA	DPV	2.5 nM–3 µM	1 nM	-	[52]
GCE/GR/Hemin/SWCNT	H_2_O_2_	CA	0.2 µM–0.4 mM	50 nM	-	[53]
CILE/3D-GR-SnO_2_/Mb	TCA	CV	5–94 mM	0.35 mM	-	[54]
SPCE/PB/CS-GO/GOD/Nf	Glucose	FIA	0.02–3.8 mM	6.7 nM	8.2 µA/mM/cm^2^	[55]
SPCE/PB/CS-GO/Lox/Nf	Lactate	FIA	1–50 mM	28 nM	0.39 µA/mM/cm^2^	
GCE/GO/GRQDs/dsDNA	DMT	DPV	1 fM–0.1 nM	1 fM		[56]
GCE/CGO/PdNPs/Nf	Paracetamol	CA	40 nM–0.8 mM	12 nM	232.89 µA/mM/cm^2^	[57]
SPCE/GO/PVFc/DAOx	Tyramine	CA	0.99 µM–0.12 mM	0.41 µM	7.99 µA/mM	[58]
SPCE/GO/PVFc/MAOx			0.99 µM–0.11 mM	0.61 µM	11.98 µA/mM	
AuE/apt/MCH/MGO	CBSK	DPV	20 to 2 × 10^6^ CFU/mL	7 CFU/mL	-	[60]
RGO-RhNPs/Lac	17β-estradiol	DPV	0.9–11 pM	0.54 pM	25.7 A/µM/cm^2^	[61]
CPE/RGO/AgNPs/ssDNA	Ba^2+^ ions	DPV	60 pM–0.80 nM & 1 nM–80 nM	45 pM	-	[62]
GCE/PEI/RGO-Fc/ChOx	Cholesterol	CA	2.5–25 µM	0.5 µM	380 mA/M/cm^2^	[63]
GCE/PEI/RGO-Fc/GOD	Glucose	CA	0.1–15.5 mM	5 µM	3.45 mA/M/cm^2^	
GCE/GNPs/RGO-Fc	BPA	DPV	5 nM–10 µM	2 nM	-	[64]
p-IL-RGO-POM/GOD	Glucose	FIA	2–20 mM	67.9 µM	14.3 µA/mM/cm^2^	[65]
p-IL-RGO-POM	H_2_O_2_	FIA	0.1–20 mM	10.2 µM	95.5 µA/mM/cm^2^	
SPCE/MWCNTs/Cyt-C	Fentanyl	ADSCV	0.5–5 µg/mL	86 ng/mL	-	[68]
AuE/CNT/p-PDA/GOD	Glucose	EIS	0.2–27.5 µM	0.2 µM	168.03 k/Ω/M	[69]
GCE/HAp5-fCNT/HRP	H_2_O_2_	CA	10–347 µM	1.91 µM	63 µA/mM	[70]
GCE/HAp20-fCNT/HRP			10–234 µM	4.4 µM	28 µA/mM	
MWCNT/IL-CS/Lac	BPA	DPV	500 nM–12 µM	8.4 nM	6.59 µA/µM	[71]
SACNTs/Urease	Uric acid	CA	100 µM–1 mM	1 µM	518.8 µA/mM/cm^2^	[72]
AuE/MWCNTs/ssDNA	Ag^+^	DPV	1 pM–10 nM	1 pM	-	[74]
PtE/CuNPs/ssDNA	MTb	DPV	1–10 nM	1 nM	42.5 µA/nM/cm^2^	[77]
AuE/FAD-GNPs	DA	DPV	0.8–8 µM	525 nM	1.25 µA/µM/cm^2^	[79]
PtE/APGNC/GOD	Glucose	CA	Up to 14 mM	23.2 µM	0.184 µA/mM	[80]
GCE/PANI/CuF/Urease	Urea	DPV	0.5–45 µM	0.17 µM	-	[81]
GCE/PANI/CoF/Urease				0.23 µM		
GCE/PANI/NiF/Urease				0.37 µM		
GCE/PANI/ZnF/Urease				0.42 µM		
GCE/ZnONPs/SPF/GOD	Glucose	CV	0.1–0.3 mM	-	52.04 µA/mM/cm^2^	[82]
CONPs/N-CNF	DA	DPV	10 nM–100 µM	9 nM	-	[83]
GCE/CeONP/HB5	HER2	EIS	1–10 ng/mL	8 pg/mL	-	[85]
GCE/BMONS/ACLE	OME	DPV	0.46 pM–0.46 µM	0.033 pM	-	[86]
	DPX		3.88 pM–3.88 µM	0.36 pM	-	
GCE/MWCNT/CSNPs/GOD	Glucose	CA	8 µM–1.5 mM	5 µM	15 mA/M/cm^2^	[88]
Nb-TDS/GOD	Glucose	CA	74.6–272.9 µM; 0.767–12.6 mM; 17.5–27.3 mM	25.7 µM	17.9 µA/mM/cm^2^	[89]
SPE/MoS_2_NSs/GNPs/BHb	CYF	DPV	4 nM–28.52 µM	5.6 nM	-	[91]
GCE/3D MGA/GOD	Glucose	FIA	2–20 mM	0.29 mM	3.36 µA/mM	[93]
SPCE/TMX-Au–PdNPs/ACLE	Paraoxon	CA	0.1–1000 µg/L	1.75 ng/L	-	[100]
GCE/MC/GNPs/TMX/ACLE	Methamidophos	DPV	1 pM–1 µM	0.134 pM	-	[102]
GCE/GRQDs/Lac	EP	CV	1–120 µM	83 nM	2.9 µA/mM/cm^2^	[108]
GCE/CRE/GRQDs/ChOx	Cholesterol	CV	16 µM–6.186 mM	5 µM	-	[109]
S-GRQDs/GNPs-CNSp/Angiopep-2	Glioma cells	EIS	100–100,000 cells/mL	40 cells/mL	-	[111]
GRQDs/TMX/PEDOT/GOD	Glucose	DPV	0–500 µM	65 µM	21.64 µA/mM/cm^2^	[112]
SPE/GOQDs/ssDNA	MiR-141	DPV	2.3–6.1 nM	91 fM	-	[113]
PBSQDs/GNSp/GOD	Glucose	DPV	0.1 µM–10 mM	1.432 nM	-	[115]
ZnSQDs/DNA	miR-200a	EIS	10 fM–1 µM	8.4 fM	374.54 Ω/M	[117]

**Table 2:** Electrocatalytic performance of various functional molecules based electrochemical biosensors.

Electrode	Analyte	Technique	Linear Range	LOD	Sensitivity	Ref.
Mg-MOF/GNPs/Mb	TCA	CV	1–200 mM	0.33 mM	-	[123]
	Nitrite	CV	0.8–18 mM	0.26 mM		
SPE/MoS_2_-Cu-MOF/GNPs/Ab	CA125	DPV	0.5 mU/mL –500 U/mL	0.5 mU	-	[124]
Cu-MOF/GNPs/CHA-HCR	MiR-21	DPV	0.1 fM–100 pM	0.02 fM	-	[125]
GCE/MMOF/GNFs	H_2_O_2_	CA	5 µM–15 mM; 15–120 mM	0.9 µM	-	[126]
GCE/FA-Zr-MOF	HeLa cells	EIS	1 × 10^2^–1 ×10^6^ cells/mL	90 cells/mL	-	[127]
GCE/NA-Zr-MOF/dsDNA	MiR-21	DPV	0.02–10 pM	8.2 fM	-	[128]
	Let-7a		0.01–10 pM	3.6 fM		
Cr-MOF/PdNPs/c-DNA	HeLa cells	DPV	5 × 10^2^–1.62 ×10^7^ cells/mL	11.25 cells/mL	-	[129]
Co-MOF/GNPs/MWCNT/Cyt-c	nitrite	DPV	5 nM–1 mM	4.4 nM	-	[131]
ITO/PANI/Cu-MOF/Ab	*E. coli*	EIS	2–2 × 10^8^ cfu/mL	2 cfu/mL	-	[132]
SPE/TCOF/SOD	Superoxide	CA	10 nM–100 µM	0.5 nM	-	[138]
GCE/COF/ACLE	Carbaryl	EIS	0.48–35 µM	0.16 µM	-	[139]
GCE/Fe_3_O_4_COF/HRP	HQ	DPV	0.5–300 µM	120 nM	-	[140]
GCE/Fe_3_O_4_COF/GNPs/DNA	ATP	CV	5 pM–50 µM	1.6 pM	-	[141]
COF/DNA/HRP	exosomes	CA	10^4^–10^7^ particles/µL	7668 particles/µL	-	[142]
GCE/AMIL/Hb	Bromate	CA	12–228 µM	3 µM	430.7 µA/mM/cm^2^	[151]
			228 µM–4.42 mM		148.4 µA/mM/cm^2^	
SPCE/ERGO/AFIL/GOD	Glucose	CA	0.05–2.4 mM	17 µM	17.7 µA/mM/cm^2^	[152]
GCE/RTAFIL/GNPs/HRP	H_2_O_2_	CA	20 µM–0.72 mM	3.7 µM	63.4 µA/mM/cm^2^	[153]
			0.72–2.77 mM		51.1 µA/mM/cm^2^	
CAA-IL/MWCNT/Lac	GA	FIA	6 µM–0.3 mM	3 µM	91.9 µA/mM/cm^2^	[154]
GCE/ASAIM-IL/GOD	Glucose	CA	1 µM–12 mM	0.572 µM	38.35 µA/M/cm^2^	[155]
SILGE/PB/TMX/GOD	Glucose	CA	0–15 mM	24.5 µM	-	[157]
GCE/AMIM-GO/CLO	CL	ADPSV	5–1000 nM	0.885 nM	-	[158]
GCE/AMIM-GO/ACLE	ACL			1.352 nM	-	
PGE/BMIM/BRC	BRCA1	DPV	2–10 µg/mL	1.48 µg/mL	1.49 µA mL/µg/cm^2^	[159]
GCE/IL-MWCNT/Catl/GOD/CS	Glucose	DPV	0.5–100 µM	0.2 µM	-	[160]
CPE/IL-GQDs/ds-DNA	Tpn	DPV	0.35–100 µM	0.1 µM	-	[161]

## Data Availability

Not applicable.

## List of Abbreviations

4-NP	4-Nitrophenol
ACL	Acetylcholine
ACLE	Acetylcholine esterase
AdSCV	Adsorptive-stripping cyclic voltammetry
AgNPs	Silver nanoparticles
AMIM TFSI-	1-Allyl-3-methylimidazolium bis(trifluoromethylsulfonyl)imide
AMQDs	Antimonene quantum dots
ASA	Aspartic acid
ATP	Adenosine triphosphate
AuE	Gold electrode
BHb	Bovine hemoglobin
BMIM HFP	1-Butyl-3-methylimidazolium hexafluorophosphate
BMIM TFB	1-Butyl-3-methylimidazolium tetrafluoroborate
BP HFP	N-Butylpyridinium hexafluorophosphate
BPA	Bisphenol A
BSA	Bovine serum albumin
CA	Chronoamperometry
Catl	Catalase
CB	Carbon black
CBSK	*Cronobacter sakazakii*
CC	Carbon cloth
CE	Counter electrode
CFU	Colony forming unit
CGO	Carboxyl graphene oxide
CHA	Catalytic hairpin assembly
ChEs	Cholesterol esterase
ChOx	Cholesterol oxidase
CILE	Carbon ionic liquid electrode
CL	Choline
CLO	Choline oxidase
C-MWCNT	Carboxylic acid functionalized multiwalled carbon nanotube
CNF	Carbon nanofiber
CNSp	Carbon nanosphere
CNTs	Carbon nanotubes
COFs	Covalent organic frameworks
CONPs	Cobalt oxide nanoparticles
CPE	Carbon paste electrode
CRE	Creasome
CRGO	Chemically reduced graphene oxide
CS	Chitosan
CSNPs	Cobalt sulfide nanoparticles
CuF	Copper ferrite
CV	Cyclic voltammetry
CVD	Chemical vapor deposition
cyc	Cysteamine
CYF	Chlorpyrifos
Cyt-c	Cytochrome c
DA	Dopamine
DAOx	Diamine oxidase
DPV	Differential pulse voltammetry
DET	Direct electron transfer
DHDP	Dihexadecylphosphate
DMT	Dimethoate
DPX	Dipterex
E. coli	Escherichia coli
EDC	1-Ethyl-3-(3-dimethylaminopropyl)carbodiimide
EIS	Electrochemical impedance spectroscopy
ERGO	Electrochemically reduced graphene oxide
FA	Folic acid
FAD	Flavin adenine dinucleotide
Fc	Ferrocene
FIA	Flow injection analysis
FLGR	Few-layered graphene
fM	Femtomolar
FR	Folate receptors
GA	Gallic acid
GCE	Glassy carbon electrode
GDY	Graphdiyne
Gld	Glutaraldehyde
GNFs	Gold nanoflowers
GNPs	Gold nanoparticles
GNSp	Gold nanosphere
GO	Graphene oxide
GOD	Glucose oxidase
GOQDs	Graphene oxide quantum dots
GR	Graphene
GRE	Graphite electrode
GRNRs	Graphene nanoribbons
GRNSs	Graphene nanosheets
GRQDs	Graphene quantum dots
H_2_O_2_	Hydrogen peroxide
HAp	Hydroxyapatite
Hb	Hemoglobin
HCR	Hybridization chain reaction
HER	Human epidermal growth factor receptor
HQ	Hydroquinone
HRP	Horseradish peroxidase
IL	Ionic liquid
ITO	Indium tin oxide
Lac	Laccase
LOD	Limit of detection
LOQ	Limit of quantification
LOx	Lactate oxidase
MAOx	Monoamine oxidase
Mb	Myoglobin
MC	Microcuboids
MCH	Mercaptohexanol
MDA	11-Mercaptoundecanoic acid
MIP	Molecularly imprinted polymer
miR	microRNA
mM	Millimolar
MNMs	Metal nanomaterials
MOFs	Metal organic frameworks
MPA	Mercaptopropionic acid
MPPy TFSI	3-Methyl-1-propylpyridinium bis(trifluoromethyl sulfonyl)imide
MPTMS	(3-Mercaptopropyl)trimethoxy silane
MTB	Mycobacterium tuberculosis
MWCNT	Multiwalled carbon nanotube
NA	Nucleic acid
Nf	Nafion
NHS	N-hydroxysuccinimide
nM	Nanomolar
NMs	Nanomaterials
NS	Nanosheets
OFWs	Organic frameworks
OME	Omethoate
PANI	Polyaniline
PB	Prussian blue
PCR	Polymerase chain reaction
PDA	Phenylenediamine
PEDOT	Poly(3,4-ethylenedioxythiophene)
PEI	Polyethyleneimine
PGE	Pencil graphite electrode
pM	Picomolar
PPI	Poly(propylene imine)
PSA	Prostate specific antigen
PtE	Platinum electrode
PVFc	Polyvinyl ferrocene
QDs	Quantum dots
RE	Reference electrode
RGO	Reduced graphene oxide
RhNPs	Rhodium nanoparticles
SCE	Saturated calomel electrode
SnDS	Tin disulfide
SPCE	Screen printed carbon electrode
SPE	Screen printed electrode
SPF	Soy protein film
SPGE	Screen printed gold electrode
SWCNTs	Single walled carbon nanotubes
TCA	Trichloroacetic acid
TCL	Thiocholine
TDS	Titanium disulfide
TMX	Ti_3_C_2_ MXenes
TPC	Terephthaloyl chloride
Tpn	Topotecan
TRGO	Thermally reduced graphene oxide
TYR	Tyrosinase
WE	Working electrode
ZnONPs	Zinc oxide nanoparticles
